# Armet/Manf and Creld2 are components of a specialized ER stress response provoked by inappropriate formation of disulphide bonds: implications for genetic skeletal diseases

**DOI:** 10.1093/hmg/ddt383

**Published:** 2013-08-15

**Authors:** Claire L. Hartley, Sarah Edwards, Lorna Mullan, Peter A. Bell, Maryline Fresquet, Raymond P. Boot-Handford, Michael D. Briggs

**Affiliations:** Wellcome Trust Centre for Cell Matrix Research, Faculty of Life Sciences, The University of Manchester, ManchesterM13 9PT, England

## Abstract

Mutant matrilin-3 (V194D) forms non-native disulphide bonded aggregates in the rER of chondrocytes from cell and mouse models of multiple epiphyseal dysplasia (MED). Intracellular retention of mutant matrilin-3 causes endoplasmic reticulum (ER) stress and induces an unfolded protein response (UPR) including the upregulation of two genes recently implicated in ER stress: *Armet* and *Creld2*. Nothing is known about the role of Armet and Creld2 in human genetic diseases. In this study, we used a variety of cell and mouse models of chondrodysplasia to determine the genotype-specific expression profiles of Armet and Creld2. We also studied their interactions with various mutant proteins and investigated their potential roles as protein disulphide isomerases (PDIs). Armet and Creld2 were up-regulated in cell and/or mouse models of chondrodysplasias caused by mutations in *Matn3* and *Col10a1*, but not *Comp*. Intriguingly, both Armet and Creld2 were also secreted into the ECM of these disease models following ER stress. Armet and Creld2 interacted with mutant matrilin-3, but not with COMP, thereby validating the genotype-specific expression. Substrate-trapping experiments confirmed Creld2 processed PDI-like activity, thus identifying a putative functional role. Finally, alanine substitution of the two terminal cysteine residues from the A-domain of V194D matrilin-3 prevented aggregation, promoted mutant protein secretion and reduced the levels of Armet and Creld2 in a cell culture model. We demonstrate that Armet and Creld2 are genotype-specific ER stress response proteins with substrate specificities, and that aggregation of mutant matrilin-3 is a key disease trigger in MED that could be exploited as a potential therapeutic target.

## INTRODUCTION

The chondrodysplasias are a clinically and genetically heterogeneous group of skeletal diseases (chondrodysplasias) that encompass over 300 different phenotypes (1). Although the clinical presentation varies from mild to lethal they are often characterized by abnormal endochondral ossification that results in disproportionate short stature. Mutations within the genes encoding a variety of cartilage extracellular matrix (ECM) structural proteins can result in numerous chondrodysplasias. These include cartilage oligomeric matrix protein (*COMP*), matrilin-3 (*MATN3*) and type IX collagen, which result in pseudoachondroplasia (PSACH: OMIN 177170) and multiple epiphyseal dysplasia (MED: OMIN 132400, 600204, 607078 and 614135) (2); and type X collagen that causes metaphyseal chondrodysplasia type Schmid (MCDS: OMIN 156500) (3).

The majority of disease-associated mutations in these genes cause misfolding of the respective proteins and their subsequent retention within the endoplasmic reticulum (ER) (4,5). Mutant protein retention results in ER stress and can lead to an UPR, whereby ER-resident proteins that are involved in protein folding are up-regulated to aid in folding and protecting the cells from stress (6,7). Other consequences of the UPR include protein degradation via ER-associated degradation (ERAD), attenuation of protein synthesis and eventually apoptosis if the misfolded protein and ER stress persist (6,8,9). Mutant protein accumulation in the ER and its subsequent degradation have been recognized as a key pathological feature in a range of different diseases, including several neurological disorders and diabetes. More recently, ER stress has been demonstrated in several mouse models of human chondrodysplasias, including PSACH-MED (10–13) and MCDS (14–16).

Matrilin-3 is the third member of the matrilin family of modular proteins and consists of a single von-Willebrand factor A-like domain (A-domain), four EGF-like motifs and a C-terminal coiled-coil oligomerization domain (17). The A-domain is arranged into a classical Rossman fold and contains a single intra-chain disulphide bond. To date, all MED causing mutations in matrilin-3 are located within the single A-domain and primarily affect residues that comprise the central β-sheet (2,18–21). A murine model of MED with the *Matn3* V194D mutation develops a progressive short-limb dysplasia resulting from decreased chondrocyte proliferation and dysregulated apoptosis. Mutant matrilin-3 is retained within the rER of chondrocytes, which leads to an UPR characterized by the upregulation of the ER-resident chaperones BiP and GRP94 (10). Microarray studies further confirmed the upregulation of numerous genes associated with ER stress and a conventional transcriptional UPR (12), including several members of the protein disulphide isomerase family A (PDIAs), specifically PDIA3, -4 and -6. The PDIAs are members of the thioredoxin superfamily of enzymes that can catalyse thiol-disulphide oxidation, reduction and isomerization and are critical for correct disulphide bond formation and/or rearrangement if incorrect (non-native) bonds are formed (22,23). There are at least 19 members of the PDIA family, and these multi-domain proteins contain at least one domain that is homologous to thioredoxin. Many of these thioredoxin domains contain a pair of active site cysteine residues (CXXC) that can shuttle between the disulphide and dithiol forms (22,24).

Interestingly, among the most highly up-regulated genes in *Matn3* V194D chondrocytes were *Armet* (arginine-rich, mutated in early stage tumours) and *Creld2* (cysteine-rich with EGF-like domains 2); two genes that have only recently been implicated in ER stress and UPR following a variety of physiological and pathological triggers (25–27).

Armet, also known as MANF (Mesencephalic Astrocyte derived Neurotrophic Factor), was identified as a gene up-regulated by various forms of ER stress in different cell lines and by cerebral ischaemia in rat (26–28). The expression of Armet was not only similar to that of the molecular chaperone BiP/GRP78, but the upregulation of Armet was also shown to be mediated by an ERSE-II element, which is frequently found in the promoters of ER chaperone genes (27). Furthermore, the over-expression of Armet in HeLa cells inhibited cell proliferation and ER stress-induced cell death (26). Interestingly, two important disease mechanisms in the *Matn3* V194D mouse include a reduction in chondrocyte proliferation and dysregulated apoptosis (10,12), suggesting an influence of Armet in the initiation and/or progression of MED.

Creld2 was recognized as an ER stress-inducible gene through microarray analysis of Neuro2a cells treated with thapsigargin. Furthermore, it has also been shown to contain a typical ER stress response element (ERSE) in its promoter region, which is positively regulated by ATF6 (25). Creld2 has been reported to mediate the intracellular trafficking of nicotinic acetylcholine receptor (nAChR) α4 and β2 subunits and is believed to be involved in the folding and assembly of α4β2 nAChRs within the ER, perhaps indicating a more general role for Creld2 in protein folding and trafficking through the ER (29).

Despite these recent descriptions, the precise roles of Creld2 and Armet remain poorly understood in human biology and genetic diseases. This current study aimed at determining the genotype-specific expression profiles of Armet and Creld2, while also investigating their interactions with mutant proteins.

## RESULTS

### Armet and Creld2 are up-regulated in cell and murine models of MED caused by matrilin-3 V194D

**Figure 1.**
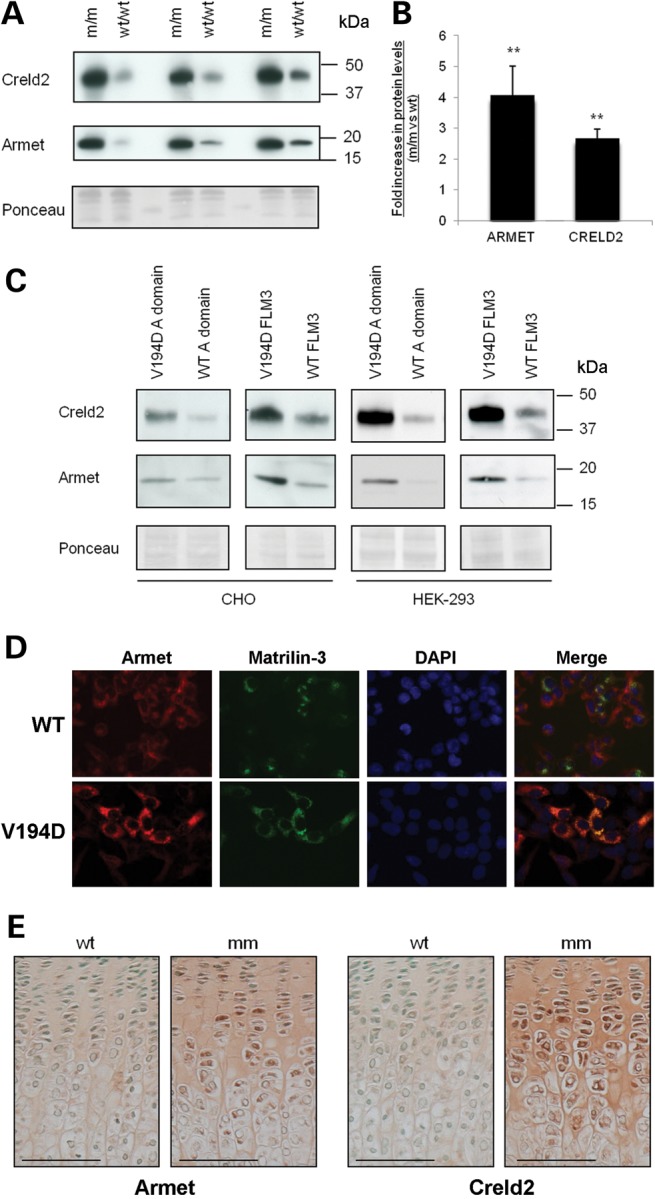
Armet and Creld2 are increased in V194D matrilin-3 mouse chondrocytes, cell culture models and growth plate cartilage. (**A**) Chondrocytes were isolated from the rib cartilage of 5-day-old *Matn3* V194D [m/m] and wild-type (WT/WT) mice. Total protein from 1 × 10^5^ cells was analysed by SDS–PAGE and western blotting using antibodies against Armet (∼18 kDa) and Creld2 (∼45 kDa). Equal protein loading was verified by Ponceau staining and three litters per genotype (∼5–10 mice pooled per litter) were analysed in three separate experiments. (**B**) Western blots were scanned and analysed by densitometry which demonstrated that there was a ∼4-fold increase in Armet and a ∼2-fold increase in Creld2 (independent *t*-test, **P < 0.01). (**C**) Cell lysate samples from CHO and HEK-293 cells expressing wild-type and V194D matrilin-3 were analysed by SDS–PAGE and western blotting and increased amounts of Armet and Creld2 were detectable in lysates from cells expressing the V194D mutation in both the full-length (FLM3) and single A-domain forms. Equal protein loading was verified by Ponceau staining. (**D**) Dual-labelling immunofluorescence microscopy confirmed that V194D matrilin-3 (green) and Armet (red) co-localized as an intracellular protein accretion (yellow/orange); DAPI was used to identify cell nuclei. (**E**) IHC using Armet and Creld2 antibodies on the tibia growth plates from 3-week-old wild-type (WT) and V194D mutant mice (mm). Chondrocytes in all zones of the mutant growth plate showed increased levels of intracellular staining for both Armet and Creld2. Interestingly, staining was also observed in the ECM of tibia growth plate cartilage. Scale bar is 100 µm; kDa = kilodaltons.

To establish the relative levels of Armet and Creld2 proteins in *Matn3* V194D mutant cartilage, sodium dodecyl sulphate-polyacrylamide gel electrophoresis (SDS–PAGE) and western blotting were performed on the intracellular proteins of chondrocytes isolated from the cartilage of new born and 5-day-old mice. Western blotting confirmed that the protein levels of both Armet and Creld2 were increased in the chondrocytes of *Matn3* V194D mice compared with wild-type controls at birth and 5 days of age (Fig. 1A, 5 days and Supplementary Material, Fig. S1, new born). Densitometry established that Armet was significantly up-regulated ∼2-fold at birth (not shown) and ∼4-fold in 5-day-old mice (Fig. 1B; **P < 0.01). Similarly, Creld2 was up-regulated ∼2-fold in newborn (not shown) and ∼3-fold in 5-day-old mutant mice (Fig. 1B; **P < 0.01). These data verified a time-dependent increase in the protein levels of Armet and Creld2, which corresponded to the gradual accumulation of mutant matrilin-3 as previously reported (10,12).

We have previously shown in cell culture models of MED that wild-type matrilin-3, either full-length protein or the single A-domain, is efficiently secreted into the culture media, whereas matrilin-3 containing MED mutations is retained intracellular (30). We therefore investigated whether the relative levels of Armet and Creld2 would also be increased in CHO and HEK-293 cell culture models expressing the p.V194D MED mutation. SDS–PAGE and western blotting demonstrated that both Armet and Creld2 were consistently increased in both the cell lines (Fig. 1C), thus confirming that the cell culture models of MED re-capitulated this characteristic pathological feature of the murine model and also demonstrating that the upregulation of Armet and Creld2, in response to mutant matrilin-3 expression, was not chondrocyte specific. Furthermore, immunofluorescent analyses confirmed that Armet (Fig. 1D) and Creld2 (not shown) were co-localized with mutant matrilin-3 in the ER.

### Armet and Creld2 are increased and secreted proteins in the growth plates of V194D matrilin-3 mice

Immunohistochemistry (IHC) was used to confirm that Armet and Creld2 were up-regulated in the growth plates of mutant mice and also to establish their precise localization within this tissue. IHC of growth plates from *Matn3* V194D mice confirmed that Armet was up-regulated and accumulated within chondrocytes of the growth plate (Fig. 1E) and around the secondary centre of ossification from 1 week of age (Supplementary Material, Fig. S2). This observation was similar to the pattern of mutant matrilin-3 retention as described previously (10,12). Surprisingly, Armet was also secreted into the ECM of growth plate cartilage of mutant mice but not wild-type controls. The increased expression of Armet and its secretion into the ECM was observed from birth (Fig. 1E and supplementary Material, Fig. S3). Similarly, IHC of growth plates from *Matn3* V194D mice showed that Creld2 accumulated within chondrocytes and its secretion into the ECM of mutant cartilage was detectable from 1 week of age (Fig. 1E and Supplementary Material, Fig. S4). These IHC data therefore support the western blotting experiments, confirming that the relative levels of Armet and Creld2 are increased within the chondrocytes of mutant mice. The accumulation of both proteins appeared to increase over time and peaked at ∼1 week for Armet, while the relative levels of Creld2 remained comparatively high over the full-time period studied.

### Armet and Creld2 are not increased in murine models of *Comp*-associated PSACH-MED but are increased in *Col10a1* MCDS

Although our studies indicated that the upregulation of Armet and Creld2 in response to the expression of *Matn3* V194D was not chondrocyte specific, we wished to determine whether their upregulation was gene product and/or mutation specific. We therefore used IHC and SDS–PAGE western blotting to study three additional mouse models of ER stress-induced chondrodysplasia; mild PSACH resulting from a mutation in the C-terminal globular domain of COMP (*Comp* T585M) (11), severe PSACH resulting from a common in-frame deletion in the type III repeat of COMP (*Comp* D469del) (13) and MCDS resulting from a NC1 mutation in type X collagen (*Col10a1* N617 K) (14).

**Figure 2.**
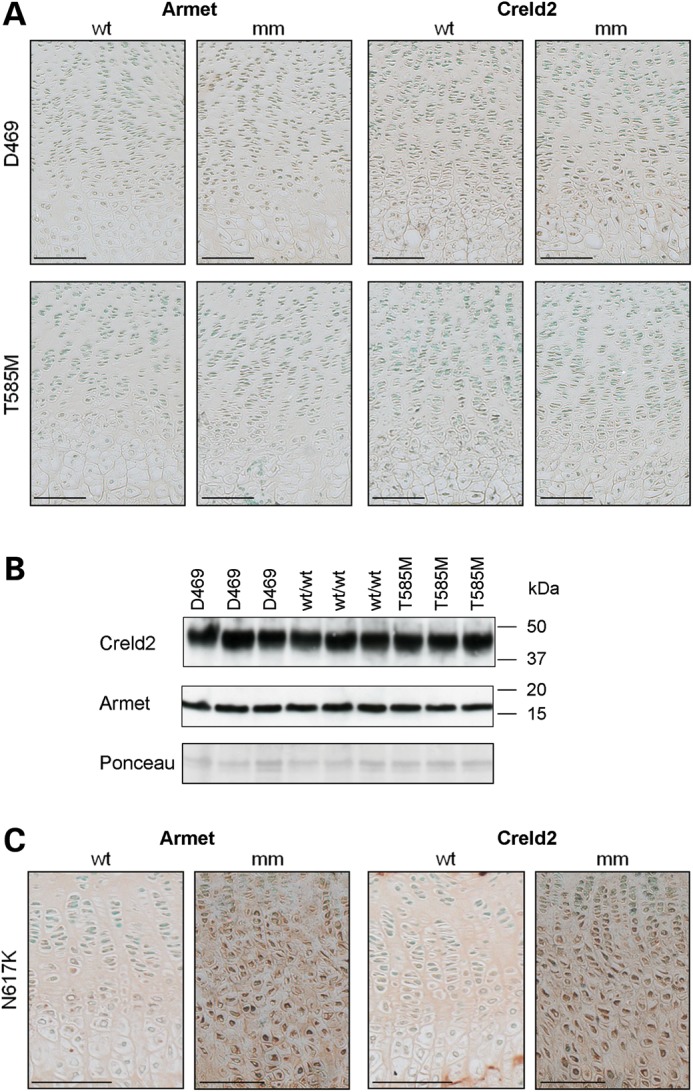
Creld2 and Armet are not up-regulated in mouse models of COMP-related PSACH-MED, but are increased in a model of MCDS. (**A**) IHC using Armet and Creld2 antibodies on tibia growth plates from 3-week-old *Comp* T585M (T585M), *Comp* D469del (D469) and matched wild-type (WT) mice. No increase in staining was observed for Armet or Creld2 in either mutant (mm) mouse model compared with wild-type (WT). (**B**) Chondrocytes were isolated from the cartilage of 5-day-old *Comp* T585M (T585M), *Comp* D469del (D469) and wild-type (WT) mice. Total protein from 1 × 10^5^ cells was loaded per lane and analysed by SDS–PAGE and western blotting. No detectable differences in the levels of Creld2 and Armet were observed in cell extracts from mutant mice (T585M and D469) compared with wild-type (WT) controls. Equal protein loading was verified by Ponceau staining. (**C**) IHC on tibia growth plates from 3-week-old *Col10a1* N617K (N617K) mice demonstrated both intracellular and ECM staining in the hypertrophic zone of growth plate cartilage. Scale bar is 100 µm; kDa = kilodaltons.

We first examined the growth plates from 1-week-old *Comp* T585M and *Comp* D469del mice by IHC and the relative levels of Armet and Creld2 remained unchanged in both mutant mice compared with their wild-type controls (Fig. 2A and Supplementary Material, Fig. S5). To confirm that there was no increase in Armet or Creld2 in the chondrocytes of these PSACH-MED models, SDS–PAGE and western blotting were performed on the intracellular proteins extracted from chondrocytes isolated from the cartilage of 5-day-old mice (Fig. 2B). Finally, we examined a mouse model of MCDS resulting from a mutation in the NC1 domain of type X collagen (*Col10a1* N617 K), and demonstrated that the relative levels of both Armet and Creld2 were increased within the chondrocytes and ECM of the hypertrophic zone (Fig. 2C). Due to the restricted expression of type X collagen in hypertrophic chondrocytes, we were unable to confirm this observation by SDS–PAGE and western blotting, but the upregulation of *Armet* and *Creld2* mRNA in *Col10a1* N617K hypertrophic chondrocytes has recently been confirmed by microarray and *in situ* in a separate study (31).

Overall, these data demonstrate that the upregulation of Armet and Creld2 is not specific to *Matn3* V194D, but is also a response to *Col10a1* N617K expression, whereas two mutant forms of COMP (T585M and D469del) do not induce increased expression.

### Matrilin-3 interacts with Armet and Creld2 in a complex with other chaperones and foldases

**Figure 3.**
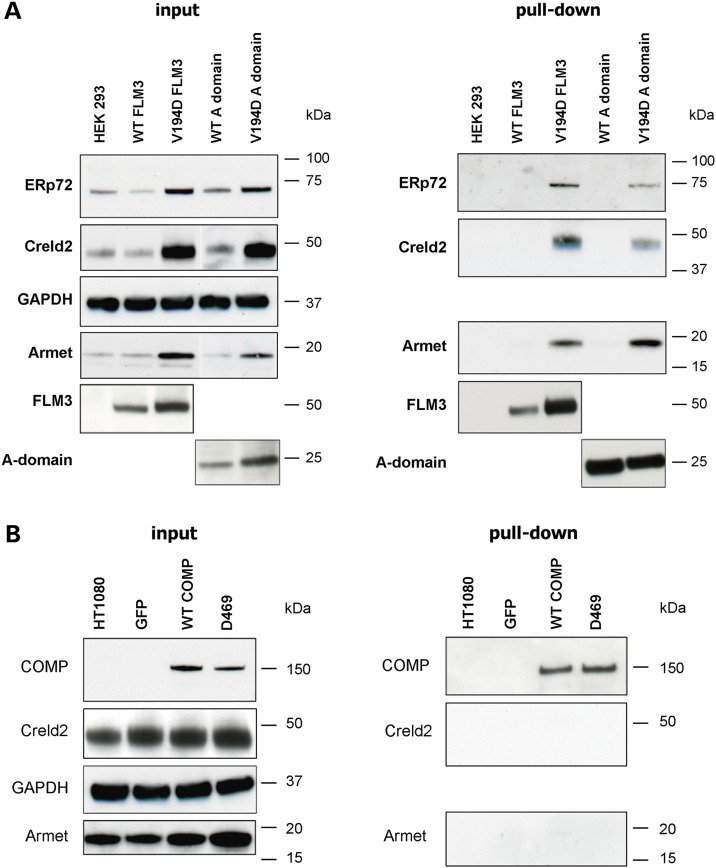
Armet and Creld2 interact in a complex with matrilin-3, but not with COMP. (**A**) Cell lysate proteins of HEK293 cells transfected with wild-type (WT) and V194D matrilin-3 expression constructs (FLAG-tagged full-length (FLM3) and the single A-domain) were immunoprecipitated with ANTI-FLAG affinity gel. SDS–PAGE and western blotting demonstrated that ERp72, Creld2 and Armet were co-precipitated with mutant matrilin-3 alone and that these interactions could be mediated by the A-domain (right panel). GAPDH confirmed equal loading and cell lysates from untransfected HEK 293 cells was used as a control. (**B**) Cell lysate proteins of HT1080 cells transfected with GFP alone or GFP-tagged wild-type COMP (WT COMP) and D469del mutant COMP (D469) expression constructs were immunoprecipitated with anti-GFP-sepharose beads. SDS–PAGE and western blotting did not identify any interactions between COMP and Armet or Creld2 (right panel). GAPDH confirmed equal loading and cell lysates from untransfected HT1080 cells was used as a control. Key: FLM3 = full-length matrilin-3; A-domain = A-domain alone comprising residues 77–263 of matrilin-3; kDa = kilodaltons.

We used co-immunoprecipitation to investigate whether both the full-length and A-domain forms of matrilin-3 could interact with Armet and Creld2, while ERp72/PDIA4, a well characterized PDI known to interact with various mutant forms of the matrilin-3 A-domain (30), was included as a control. These experiments confirmed that full-length mutant matrilin-3 interacted with Armet, Creld2 and ERp72/PDIA4; while the use of mutant A-domain alone refined the site of these interactions to this specific region (Fig. 3A).

Proteomic interrogation by liquid chromatography-mass spectrometry (LC-MS)/MS of the Flag-precipitated protein complexes revealed that a number of different chaperones and foldases were also present in both the wild-type and mutant matrilin-3 protein complexes (Table 1). These included BiP/GRP78, GRP94, PDI (PDIA1, -3, -4 and -6) and peptidylprolyl isomerases A and B; however, there were clearly more peptides detected for PDIA4, PDIA6, BiP/GRP78 and GRP94 in the mutant protein samples. Finally, the only protein that was almost entirely absent from wild-type matrilin-3 complexes, but was present in significant quantities in mutant protein complexes of both the A-domain and full-length matrilin-3, was hypoxia up-regulated protein 1 (HYOU1/ORP150/GRP170), which is a protein implicated in ER stress and several human diseases (32).

**Table 1.** Summary of the results of spectral counting from FLAG and GFP-precipitated complexes of matrilin-3 and COMP, respectively

Gene	Type	293	WT FLM3	V194D FLM3	WT A-domain	V194D A-domain	1080	WT COMP	D469del COMP
PDIA1	PDI	0/0	4/6	8/7	6/3	8/8	0/0	7/8	6/5
PDIA3	PDI	0/0	3/0	4/0	3/0	6/7	0/0	0/0	0/0
PDIA4	PDI	0/0	3/3	22/24	5/3	13/7	0/0	8/5	9/11
PDIA6	PDI	0/4	9/4	11/10	8/5	10/10	0/0	0/0	0/0
PPIA	PPI	0/3	2/2	0/0	0/2	3/0	0/3	0/2	0/0
PPIB	PPI	0/0	4/3	4/7	5/0	5/5	0/0	3/3	7/5
GRP78	Chaperone	3/8	14/16	50/46	14/8	52/43	0/10	26/20	27/29
GRP94	Chaperone	0/4	9/3	32/20	9/6	22/19	4/2	19/23	21/15
ORP150	Chaperone	0/0	0/0	10/12	2/0	10/10	0/0	0/0	4/2
DNAJC3	Co-Chaperone	0/0	0/0	0/2	0/0	3/0	0/0	0/0	0/0
DNAJC10	Co-Chaperone	0/0	0/0	2/3	0/0	2/3	0/0	0/0	0/0
MATN3	ECM protein	0/7	15/12	26/28	37/9	24/15	–	–	–
COMP	ECM protein	–	–	–	–	–	0/0	26/18	32/12

Total protein pools following FLAG and GFP precipitation were analysed by LC-MS/MS and the data evaluated using Mascot against the UniProt human database and validated with Scaffold using peptide/protein confidence values of 0.95 and 0.99, respectively. Positively identified proteins were defined as those having a number of matched peptide spectra >2. Two biological replicates were used in all experiments and the number of matched spectra from each experiment is shown separated with a forward slash (/).

Key: 293 = untransfected HEK293 cells; 1080 = untransfected HT1080 cells; WT = wild type; V194D = mutant matrilin-3; D469del = mutant COMP; FLM3 = full-length matrilin-3; A-domain = matrilin-3 A-domain alone; PDI = protein disulphide isomerase; PPI = peptidylprolyl isomerase; ECM = extracellular matrix protein.

### Armet and Creld2 are not present in a complex with COMP and other chaperones

Following co-immunoprecipitation, we did not detect any interactions between COMP (wild type or D469del) and either Armet or Creld2 (Fig. 3B). LC-MS/MS of the GFP-precipitated protein complexes confirmed that wild-type COMP formed complexes with a variety of chaperones and foldases including PDIAs (PDIA1 and -4), BiP/GRP78, GRP94 and peptidylprolyl isomerase B (PPIB) (Table 1). However, unlike *Matn3* V194D there were no quantitative differences in the proteomic portrait of D469del mutant COMP compared with wild-type, with the possible exception of hypoxia up-regulated protein 1 (HYOU1), which was only detected in mutant COMP samples.

### Increased expression of Armet and Creld2 is associated with a specific increase in PDIs in *Matn3* and *Col10a1* disease models

We have previously shown by microarray analysis that a UPR is initiated through the expression of *Matn3* V194D (12). Interestingly, of the 20 most significantly up-regulated genes, three were members of the PDIA protein family, namely *Pdia3*/*Erp57*, *Pdia4*/*Erp72* and *Pdia6/P5*, while PDI was shown to be up-regulated by western blotting. These expression data are therefore consistent with the proteomic profile of the co-precipitated mutant protein complexes.

In order to gain insight into why Armet and Creld2 were only increased in the murine models of *Matn3*-MED and *Col10a1*-MCDS, and not the *Comp* models of PSACH-MED, we compared the relative expression levels of *Armet*, *Creld2* and members of the PDIA protein family in chondrocytes from the following mouse models; *Matn3* V194D (12), *Comp* D469del (13), *Comp* T585M (K Pirog personal communication) and *Col10a1* N617K (31) (Table 2). It is noteworthy that there was a consistent and significant upregulation of several PDIAs in the *Matn3* V194D and *Col10a1* N617K mutant mice, whereas there was no such upregulation in the *Comp* D469del and *Comp* T585M mice. These expression data and the co-immunoprecipitation studies (Fig. 3) led us to consider whether Armet and/or Creld2 possessed PDI-like activities.

**Table 2.** PDIs are up-regulated in mouse models of Matn3 MED and Col10a1 MCDS but not Comp PSACH-MED

Gene and/or protein name	V194D d5	D469del d5	T585M d5	N617K NB
Creld2	5.77	–	1.30	7.66
Armet/MANF	4.29	−1.33	1.24	2.99
PDIA1/P4HB	1.57	–	−1.03 (ns)	2.49
PDIA2/PDIP	1.42 (ns)	–	1.02 (ns)	–
PDIA3/ERp57/GRP58	2.03	1.12	1.24	2.05
PDIA4/ERp72	3.16	1.76 (ns)	1.22	16.37
PDIA5/PDIR	1.34	1.07	−1.09 (ns)	1.62 (ns)
PDIA6/P5	3.18	−1.09	1.27	4.97

Selected gene expression profiles of chondrocytes from 5-day-old (or new born for *Col10a1*) wild-type and the mutant mice genotypes *Matn3* V194D (12), *Comp* D469del (13), *Comp* T585M (unpublished) and *Col10a1* N617K (31) were compared. Gene expression levels were previously determined by microarray analysis as described in relevant papers. The relative expression levels of Armet, Creld2 and PDIAs1-6 are represented as fold change (mutant versus wild type), and all values are significant unless otherwise stated (ns = not significant). Significant upregulation was determined by PPLR values of >0.95.

Key: d5 = 5-day-old; NB = new born.

### Creld2 possesses putative PDI-like activity whereas Armet does not

Armet has recently been proposed to be a putative PDIA due to the presence of a CXXC motif in the C-terminal domain (22,33), which is a common feature of thiol/disulphide oxidoreductases (22). Similarly, Creld2 has a number of CXXC motifs, which may indicate that it also possesses isomerase activity. In order to investigate this potential function, we generated a series of Armet and Creld2 substrate-trapping mutants similar to those previously generated for other PDIAs (e.g. ERp72, ERp57, ERp46 and PDI), which have all previously been validated and characterized in depth (34). Following sequence alignment comparisons between Creld2, Armet and PDI, we identified potential amino- and carboxyl-terminal CXXC motifs (Supplementary Material, Fig. S6). For each substrate-trapping construct, we used *in vitro* mutagenesis to convert the second cysteine of each selected CXXC motif into alanine, thereby generating eight constructs, which included wild-type and double N- and C-terminal mutants (Supplementary Material, Fig. S7). HT1080 cells were then transfected with wild-type or mutant (N-CXXA, C-CXXA and N/C-CXXA) constructs and selection with G418 allowed stable cell lines to be established. Once confluent, and following treatment with NEM to prevent post-lysis thiol exchange, the total cell lysate proteins were separated by SDS–PAGE and proteins were detected by western blotting with an anti-V5 antibody. We also included as a positive control in this experiment the previously characterized ERp72 substrate-trapping mutant as an example of the typical profile that would be expected from a confirmed PDI (34). In this definitive study, the authors showed unequivocally that substrate-trapping mutants of ERp57 (PDIA3), PDI (PDIA1), P5 (PDIA6), ERp18 (PDIA6), ERp72 (PDIA4) and ERp46 (PDIA15) all formed higher order mixed disulphides, whereas the repsective wild-type proteins did not (34). We therefore included in our experiments only the substrate-trapping mutant form of ERp72 (and not the wild-type protein) as a visual example for the formation of higher order mixed disulphides.

**Figure 4.**
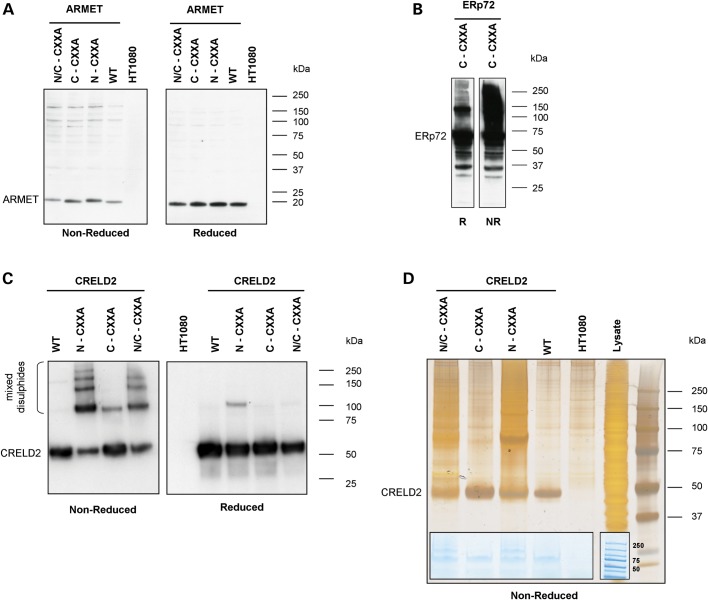
Creld2 processes putative PDI-like activity, whereas Armet does not. Total cell lysate proteins from HT1080 cells stably transfected with Armet-V5, Creld2-V5 or ERp72-V5 substrate-trapping mutants were separated by SDS–PAGE and analysed by western blotting with an anti-V5 antibody. (**A**) There was no evidence of higher order mixed disulphides formed between putative substrate proteins and either wild-type Armet (WT) or the individual substrate-trapping mutants (N/C-CXXA, C-CXXA and N-CXXA). (**B**) In comparison, the control ERp72 substrate-trapping mutant (C-CXXA) demonstrated the formation of mixed disulphides with substrate proteins. (**C**) Both the N-CXXA and N/C-CXXA substrate-trapping mutants of Creld2 formed high-molecular weight mixed disulphides (left panel) that were resolved on reduction (right panel). In contrast, wild-type Creld2 (WT) and the C-CXXA trapping mutant did not form higher molecular weight aggregates with putative substrate proteins. (**D**) V5 co-immunoprecipitated proteins from the various Creld2 substrate-trapping cell lines were resolved by SDS–PAGE and viewed by silver staining or instant blue (insert). Total protein pools >50 kDa were excised from each lane of the instant blue gel for liquid chromatography-mass spectrometry/MS analysis. Key: N/C-CXXA = amino and carboxyl terminal double substrate-trapping mutant; C-CXXA = carboxyl terminal substrate-trapping mutant; N-CXXA = amino terminal substrate-trapping mutant; WT = wild- type Armet or Creld2; HT1080 = untransfected HT1080 cells; lysate = total protein lysate prior to V5 co-immunoprecipitation; R = reduced protein samples; NR = non-reduced protein samples; kDa = kilodaltons.

All the three substrate-trapping mutants of Armet (N-CXXA, C-CXXA and N/C-CXXA) had a similar profile to the wild type (under both reducing and non-reducing conditions), which suggested that no higher order mixed disulphides had been formed with substrate proteins (Fig. 4A), whereas the ERp72 substrate-trapping mutant clearly showed the formation of mixed disulphide complexes (Fig. 4B), which were consistent with the previously published profile (34). Similar to ERp72, the mutation of putative active sites in Creld2 allowed the formation of a number of higher order complexes containing Creld2-V5 (Fig. 4C, left panel). These complexes were disrupted following reduction by dithiothreitol (DTT), confirming that mixed disulphide bonds had formed (Fig. 4C, right panel). Furthermore, there was specificity in which CXXC motif was mediating the interaction(s). For example, only the N-CXXA and N/C-CXXA proteins formed high-molecular weight mixed disulphides, indicating that it was the amino-terminal CXXC motif that possessed the isomerase activity. To identify the individual substrates forming mixed disulphide complexes with Creld2, we performed LC-MS/MS after affinity isolation of the protein complexes using anti-V5 agarose beads (Fig. 4D and Table 3). This analysis, which was performed in HT1080 cells, identified laminin-5 β3 (LAMB3), collagen α1(VI) and α3(VI), thrombospondin-1 and integrin α-3 as potential substrates, while BiP/GRP78, GRP94 and PDIA1, -3 and -6 were also present within these complexes.

**Table 3.** Creld2 is part of mixed disulphide complexes with structural proteins, chaperones and PDIs

Protein	Type	HT1080	Creld2 WT	Creld2 N-CXXA	Creld2 C-CXXA	Creld2 N/C-CXXA
BiP	Chaperone	0/0	4/0/6	16/17/19	3/0/9	17/16/17
GRP94	Chaperone	0/0	0/0/3	0/11/3	0/0/2	8/8/9
HSP90β	Chaperone	4/0	4/0/4	10/5/10	0/0/4	17/5/17
HSPA8	Chaperone	0/0	6/0/3	7/13/9	2/0/4	14/5/14
SERPINH1	Chaperone	0/0	0/0/0	2/6/2	0/0/0	4/3/3
PDIA1	PDI	0/0	3/0/0	3/6/4	0/0/4	7/6/5
PDIA3	PDI	0/0	0/0/0	3/6/5	0/0/7	12/11/8
PDIA4	PDI	0/0	0/0/0	0/3/0	0/0/0	7/4/4
LAMB3	ECM protein	0/0	0/0/0	11/15/22	2/0/14	14/13/17
α1(VI) chain	ECM protein	0/0	0/0/0	7/9/15	0/0/0	9/5/5
α3(VI) chain	ECM protein	0/0	0/0/0	3/9/22	0/0/14	19/15/12
TSP1	ECM protein	0/0	0/0/0	14/11/23	0/0/5	6/0/7
Integrin alpha-3	Receptor	0/0	0/0/0	6/4/14	0/0/0	9/6/10
Pyruvate kinase isozymes M1/M2	Glycolytic enzyme	0/0	0/0/9	10/11/13	0/0/5	11/5/16

A summary of the proteins identified by spectral counting from V5-precipitated complexes of the Creld2 substrate-trapping mutants. Total protein pools were analysed by LC-MS/MS and the data analysed using Mascot against the UniProt human database and validated with Scaffold using peptide/protein confidence values of 0.95 and 0.99, respectively. Positively identified proteins were defined as those having a number of matched peptide spectra greater than two. Three biological replicates were used in all experiments and the number of spectra from each experiment is separated with a forward slash (/).

Key: N/C-CXXA = amino and carboxyl terminal double substrate-trapping mutant; C-CXXA = carboxyl terminal substrate-trapping mutant; N-CXXA = amino terminal substrate-trapping mutant; WT = wild-type Creld2; HT1080 = untransfected HT1080 cells; PDI = protein disulphide isomerase; PPI = peptidylprolyl isomerase; ECM = extracellular matrix.

### N-CXXA Creld2 substrate-trapping mutant shows specificity for mutant matrilin-3

**Figure 5.**
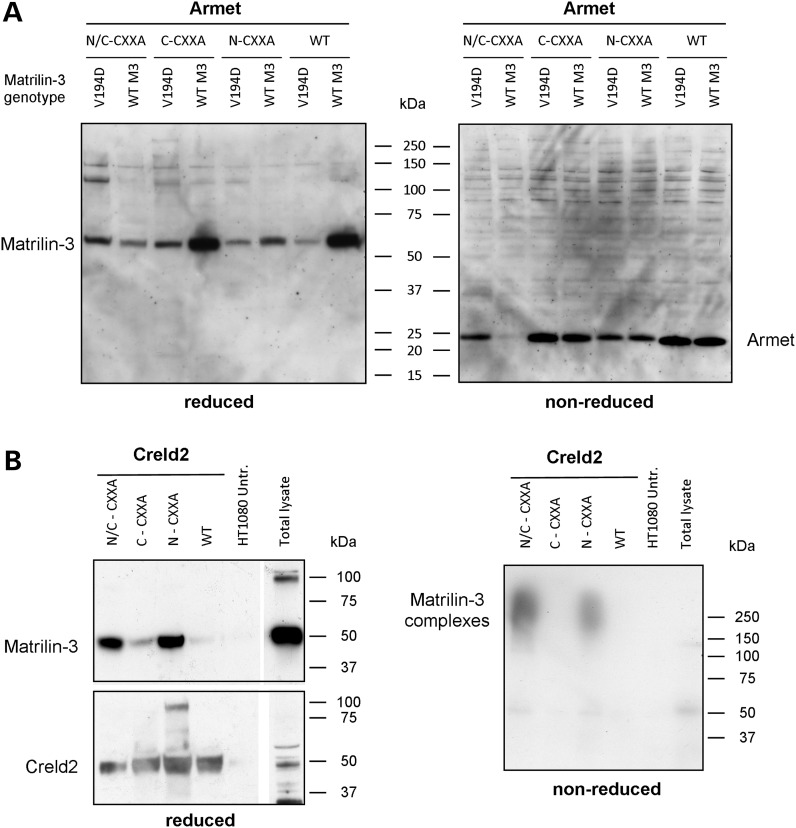
N-CXXA Creld2 substrate-trapping mutant shows specificity for mutant matrilin-3. Wild-type Armet-V5 and Creld2-V5 (WT) and the various substrate-trapping (N/C-CXXA, C-CXXA and N-CXXA) cell lines were co-transfected with wild-type (WT M3) or V194D mutant (V194D) matrilin-3 expression constructs. (**A**) Reducing SDS–PAGE and western blotting (anti-FLAG) on total cell lysate proteins confirmed the co-expression of WT and V194D matrilin-3 in all Armet cell lines (left panel: WT Armet and the various substrate-trapping lines). However, non-reducing SDS–PAGE and western blotting for Armet (anti-V5) did not detect any higher order mixed disulphides in all substrate-trapping and wild-type cell lines (right panel). (**B**) Co-immunoprecipitation with V5 followed by reducing SDS–PAGE and western blotting for Creld2 (anti-V5) and matrilin-3 (anti-FLAG) confirmed interactions between full-length V194D matrilin-3 and only the N/C-CXXA and N-CXXA substrate-trapping cell lines (left panel). When these samples were run under non-reducing conditions, the presence of matrilin-3 (anti-FLAG) containing higher order mixed disulphide complexes was demonstrated (right panel). Total cell lysate proteins from HT1080 cells (either untransfected or transfected with WT Creld2) were used controls. Key: WT M3 = wild-type matilin-3; V194D = V194D mutant matrilin-3; N/C-CXXA = amino and carboxyl terminal double substrate-trapping mutant; C-CXXA = carboxyl terminal substrate-trapping mutant; N-CXXA = amino terminal substrate-trapping mutant; WT = wild-type Armet or Creld2; HT1080 = untransfected HT1080 cells; kDa = kilodaltons.

Since HT1080 cells do not express cartilage structural proteins, we wished to test directly whether Armet or Creld2 substrate-trapping mutants could capture mutant matrilin-3. As expected co-transfection of the Armet HT1080 substrate-trapping cell lines with WT and V194D matrilin-3 expression constructs (Fig. 5A; left panel) failed to identify any mixed disulphides (Fig. 5A; right panel), thereby confirming that Armet most likely formed non-covalent interactions with matrilin-3 protein-folding complexes (see Fig. 3A). In contrast, the N-CXXA and N/C-CXXA substrate-trapping mutants of Creld2 were able to capture full-length mutant matrilin-3 (Fig. 5B; left panel) that existed as heterogeneous higher order mixed disulphide complexes under non-reducing conditions (Fig. 5B; right panel). This finding was consistent with the previous experiment (Fig. 4C) and confirmed the location of the isomerase activity of Creld2 to the amino terminal CXXC motif. However, we also noted that the C-CXXA mutant was able to interact to a small extent with mutant matrilin-3, suggesting that there may be some limited activity at this CXXA site of Creld2.

### Substitution of the two terminal cysteine residues from the A-domain of V194D matrilin-3 prevents aggregation, promotes mutant protein secretion and reduces the levels of Creld2 and Armet

**Figure 6.**
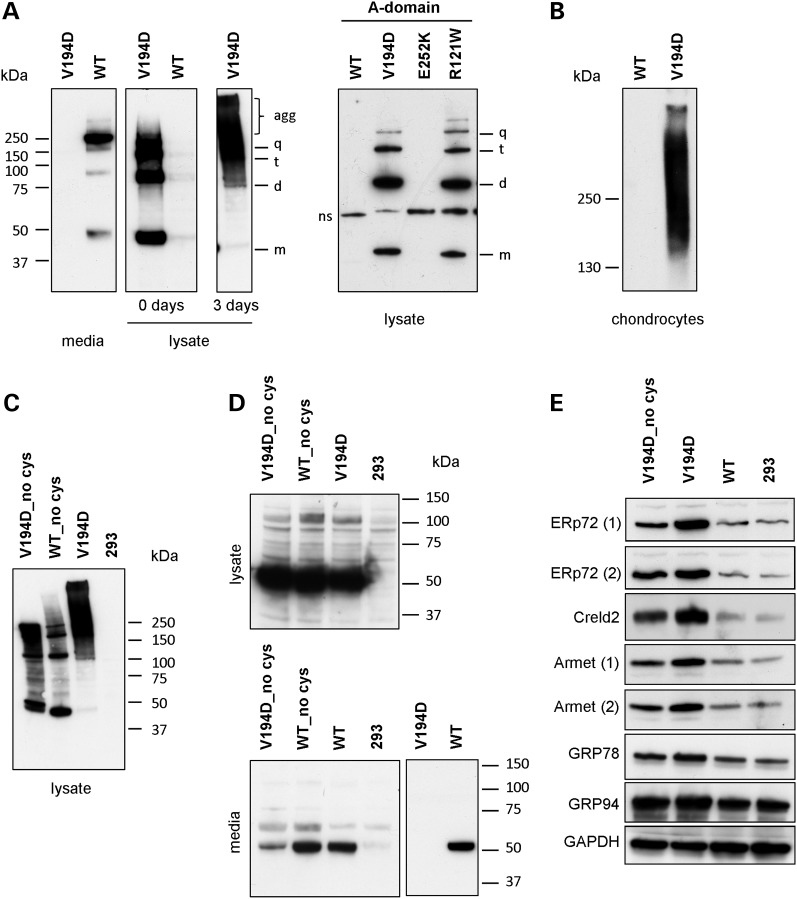
Mutant matrilin-3 forms non-native disulphide bonded aggregates that can be resolved by deletion of the A-domain terminal cysteine residues. SDS–PAGE and western blot analysis confirmed the intracellular retention and aggregation over time (*t* = day 0 or day 3 following confluency after transfection) of V194D mutant matrilin-3 (V194D) in the cell lysate of (**A**) cellular models or (**B**) isolated mouse chondrocytes. (A; left panel) demonstrates that only wild-type (WT) matrilin-3 is secreted into the culture medium predominately as a tetramer, whereas as V194D mutant matrilin-3 (V194D) is retained initially as various oligomeric forms (*t* = 0), but these aggregate over time (*t* = 3) to form high-molecular weight aggregates. (A; right panel) confirms that aggregation is mediated through the A-domain by the presence of various oligomeric forms (1x to 5x). V194D and R121W are typical *MATN3*-MED mutations, whereas E252K is a known polymorphism and is comparable to wild type (WT). (**C**) *In vitro* substitution of the terminal cysteine residues of the A-domain (V194D_no cys) resolved mutant protein aggregation (V194D) and did not affect oligomerization of either the wild-type (WT_no cys) or mutant proteins when analysed by non-reducing SDS–PAGE and western blotting (anti-FLAG). (**D**) Substitution of the terminal cysteine residues with alanine promoted secretion of the mutant V194D protein (V194D-no cys) as visualized by reducing SDS–PAGE. Furthermore, (**E**) there was also selective reduction in the levels of ERp72, Creld2, and Armet, but not GRP78 or GRP94. Ponceau staining (not shown) and GAPDH were used as loading controls. Key: WT = wild-type matrilin-3; V194D = mutant matrilin-3; V194D_no cys & WT_no cys = matrilin-3 with cysteine residues 77 and 263 replaced with alanine (mutant and wild-type respectively); 293 = cell lysate from untransfected HEK293 cells; agg = non-native disulphide bonded aggregates of mutant matrilin-3; q = tetramer, *t* = trimer, d = dimer and m = monomers of matrilin-3; ns = non-specific band; kDa = kilodaltons. All gels show full-length matrilin-3 unless stated otherwise and the number of replicates in (E) is indicated.

The expression of V194D matrilin-3 consistently results in aggregation through non-native disulphide bonds and intracellular retention in cell and mouse models of MED (Fig. 6A and B, respectively) (12,30,35). Moreover, this aggregation is mediated through the mutant A-domains alone (Fig. 6A; right panel) and can be resolved to a single molecular form on reduction (21,30) (and not shown). We therefore tested the hypothesis that alanine substitution of the two terminal cysteine residues (Cys77 and Cys263) from the A-domain of V194D matrilin-3 would reduce mutant protein aggregation, promote the secretion of a proportion of V194D matrilin-3 and influence the expression of PDIs.

The removal of both cysteine residues prevented the formation of V194D matrilin-3 higher order disulphide-bonded aggregates (Fig. 6C), but did not prevent the folding and secretion of wild-type matrilin-3 A-domain. Moreover, this genetic manipulation also promoted to some extent the secretion of V194D matrilin-3 (Fig. 6D) and selectively reduced the protein levels of ERp72, Creld2 and Armet, but not GRP78 and GRP94 (Fig. 6E).

## DISCUSSION

This study demonstrates for the first time the genotype-specific upregulation and secretion of Armet and Creld2 in various cell and mouse models of genetic skeletal diseases. Moreover, we have demonstrated that both Armet and Creld2 have substrate specificity in their binding to structural proteins and that Creld2 possess PDI-like activity. This is the first study to identify a potential role for both Armet and Creld2 and to confirm that they are key markers in human ER stress-related diseases. Finally, we present data that the aggregation of mutant matrilin-3 through non-native disulphide bonds between the A-domains is a key disease trigger that promotes specialization of the UPR toolbox (36).

Perhaps surprisingly, we observed that both Armet and Creld2 were present within the cartilage ECM of mutant mouse growth plates, confirming that these proteins can be secreted under certain pathological conditions. Many ER resident proteins are prevented from being secreted via an ER retention motif (KDEL) at their extreme C-terminus, which binds with high affinity to KDEL receptors (KDEL-R) located in the intermediate compartment or in the cis-Golgi (37). Interestingly, both Armet and Creld2 contain putative ER retention motifs at their C-terminus (RTDL and REDL, respectively) (38), suggesting that these proteins might remain resident in the ER. However, while previous reports have shown that both Armet and Creld2 are primarily retained within the ER or Golgi, they can also be secreted into cell culture media under certain experimental conditions (25,26,39). For example, these proteins can be secreted if the putative ER retention signals are disrupted, either by deletion or having a peptide tag engineered at the C-terminus (26,27,39). In other experiments, the ER retention motif has not been disrupted but cells have been subjected to ER stress by chemical agents such as tunicamycin or by culturing cells in serum-free medium. Overall, these studies have suggested that Armet and Creld2 can be secreted *in vitro* during different ER stress conditions. Since Armet and Creld2 are only detectable at significant levels in the ECM of *Matn3* V194D and *Col10a1* N617K mutant growth plates and not in wild-type controls, our study demonstrates for the first time that both proteins are up-regulated and secreted during ER stress in gene-targeted disease models. This observation is consistent with the hypothesis that under normal physiological conditions, Armet and Creld2 are able to bind to the KDEL-R and remain within the ER (40); however, it is believed that KDEL-variants bind with lower affinity than KDEL itself (38). During ER stress, the relative levels of Armet and Creld2 are increased, along with other UPR-related proteins such as BiP and GRP94, while the relative expression of the three mammalian KDEL receptors (KDELR1–3) are not increased (41). Indeed, microarray analysis confirmed that the relative levels of *Kdelr1*, *Kdelr2* and *Kdelr3* in V194D mutant compared with control chondrocytes were −1.22, 1.43 and 1.28, respectively (not shown). Therefore, upon activation of the UPR proteins such as BiP, which contain perfect ER retention motifs that have higher affinity for the KDEL receptor, would compete with proteins which contain a motif that has a lower binding affinity, thereby allowing the latter proteins to escape the ER and ultimately be secreted. The interesting observation that Armet and Creld2 are secreted under conditions of increased ER stress suggests the possibility that they may be exploited as soluble extracellular biomarkers of ER stress-related diseases that are gene product and/or mutation specific.

Substrate-trapping experiments demonstrated that Creld2 possesses PDI-like activity and in addition to matrilin-3, we also identified laminin-5 β3, collagen VI and thrombospondin-1 (TSP1) as potential substrates. All three proteins have numerous intramolecular disulphide bonds. Indeed, the α1(VI) and α3(VI) chains contain up to three and seven VWA domains, respectively (42), which are very similar in structure to the one found in matrilin-3, while laminin-5 β3 has numerous intermolecular disulphide bonds in various domains and also forms disulphide bonded trimers with the α3 and γ2 chains. TSP1 is the archetypal member of the TSP protein family, which includes COMP (TSP5), but has additional domains not shared with COMP such as the type 1 repeats and procollagen homology domain. Mutations in the VWA domains of type VI collagen result in Bethlem and Ulrich muscular dystrophies (42), while mutations in LAMB3 cause junctional epidermolysis bullosa (both Herlitz and non-Herlitz type) (43). In both diseases, ER stress and an UPR have not been investigated as potential mechanisms, although mutations in type VI collagen have been shown to cause impaired secretion and mutant protein retention (44,45). The role of ER stress and UPR specialization in these connective tissues diseases, and in particular whether ARMET and Creld2 are up-regulated, warrants further investigation in order to delineate a range of different phenotypes that may share a common disease trigger.

The co-immunoprecipitation experiments were also consistent with the recently published interaction map of endoplasmic reticulum chaperones and foldases (36). For example, we noted that matrilin-3 formed ER multi-protein complexes with BiP/GRP78, GRP94, selected PDIAs and PPIs. Indeed, the PPIB (cyclophilin B) multi-protein composite containing PDIA1, PDIA4/ERp72, PDIA6/P5, BiP/GRP78 and GRP94 was found with matrilin-3 A-domain complexes (Table 1), while the calnexin cycle components (CANX and CALR) were absent, which supports the separation of these two systems in this disease model (36). Moreover, there were increased levels of these proteins in mutant matrilin-3 complexes, which was consistent with our previous gene expression studies (Table 2). Wild-type COMP also showed preference for the PPIB multi-protein complex, but in contrast there was no increase in these components with D469del mutant COMP, confirming our previous finding of a lack of transcriptional UPR with this mutation (13). Finally, the interesting observation that HYOU1 was almost exclusively associated with mutant V194D matrilin-3, and to a lesser extent D469del COMP, highlights an important role for this protein in binding to mutant protein substrates and its cytoprotective role warrants further investigation in MED disease models (32).

Overall, our data suggest that the upregulation of Armet and Creld2 with the PPIB ER-folding complex may not simply be a consequence of UPR activation but could be part of a specialized UPR that is tailored for certain misfolded proteins that expose an unpaired cysteine residue. Indeed, Armet has recently been suggested to facilitate the formation of cysteine bridges and protein folding in the ER during neurodegenerative diseases (33), while a C96Y mutation in insulin leads to the disruption of intramolecular disulphide bonds (46), and the expression of this mutation in pancreatic cell lines leads to induction of *Armet* and *Creld2* along with various PDIAs (47).

The upregulation of various PDIAs in the *Matn3* V194D mutant chondrocytes might be expected since mutant matrilin-3 forms misfolded aggregates, both *in vivo* and *in vitro*, which dissociate upon reduction, indicating that these complexes form via non-native disulphide bonds. Furthermore, mutant matrilin-3 A-domains have also been shown to directly interact with PDIA4/ERp72 in a cell culture model of MED (30). Similarly, although expected to form stable type X collagen homotrimers, certain mutant forms of type X collagen have been shown to form an unusual dimer, which also dissociates upon reduction (48). The formation of this mutant type X collagen dimer, via a non-native disulphide bond, is believed to result from a dramatic conformational change since the sulfhydryl group in the NC1 domain is not solvent-exposed in the correctly folded α1(X) trimer. Finally, several mutant forms of type X collagen have been shown to co-immunoprecipitate with PDI (49); however, additional experimentation is required to define the role of Armet and Creld2 in MCDS.

Conversely, there is no evidence to support non-native disulphide bond formation and protein aggregation due to mutations in *COMP*. For example, cell culture studies on COMP with p.D469del, p.D472Y or p.D475N mutations confirmed impaired secretion of the mutant protein, but also demonstrated that both retained and secreted mutant COMP forms pentameric molecules similar to the wild-type protein (50–52). Overall, these studies demonstrate that specific mutations in the type III repeat region of COMP do not affect its ability to form pentamers, suggesting that these mutations do not cause a dramatic conformational change of the protein that might leave unpaired cysteine residues.

We hypothesized that the formation of non-native disulphide bonded aggregates of mutant V194D matrilin-3 was the direct result of delayed or incomplete folding of the central β-sheet of the A-domain that results in prolonged exposure of the thiol groups of the two terminal cysteine residues (Cys77 and Cys263). This in turn promotes the formation of intermolecular disulphide bonds between matrilin-3 oligomers that cause mutant protein aggregation and render these complexes resistant to retro-translocation and ERAD. To test this hypothesis, we engineered expression constructs in which the two terminal cysteines were replaced by alanine residues. In the first instance, this proof-of-principle experiment demonstrated that removal of Cys77 and Cys263 did not disrupt to any great extent the folding and secretion of wild-type matrilin-3. This result in itself was not surprising since some vWFA domains and related integrin I-domains do not possess these terminal cysteine residues (53). In contrast, we saw a dramatic effect on the aggregation and secretion of mutant matrilin-3. For example, deletion of Cys77 and Cys263 completely abolished the formation of disulphide-bonded high-molecular weight aggregates and promoted the secretion of a proportion of V194D matrilin-3. Moreover, this resulted in a reduction of specific chaperones and foldases including Armet, Creld2 and ERp72, thus confirming their direct role in mediating disulphide bond formation in this disease model.

In conclusion, this study has discovered that the aggregation of mutant matrilin-3 is a key disease trigger in this form of MED, and that the prevention of this protein accretion, by either enhancing the folding with corrector-molecules (54,55) or preventing non-native disulphide bond formation by blocking the unpaired cysteine residues (56), offers novel therapeutic targets for further validation.

## MATERIALS AND METHODS

### Transgenic mice models of human genetic skeletal diseases

*Matn3* V194D, *Comp* T585M mice, *Comp* D469del mice and C*ol10a1* N617K mice were generated as previously described (10,11,13,14). Mice homozygous for the respective mutations were compared with their wild-type counterparts for these studies.

### Matrilin-3 and COMP cellular models of PSACH-MED

Complementary DNAs encoding full-length matrilin-3 (FLM3) and the A-domain alone (both wild type and p.V194D) had previously been cloned into pCMV-Tag4 (FLM3) and pSecTag2A (A-domain) vectors with C-terminal C-MYC or FLAG epitope tags, respectively, and used to establish stable CHO cell lines (30). These cDNAs were also subcloned into the expression vector pCEP4 and transfected into HEK 293 cells as described previously (21). Expression constructs encoding wild-type COMP and the D469del mutation had previously been cloned into pEGFP-N3 with a C-terminal GFP tag and transfected into HT1080 cells (13). Mutation of the terminal cysteines (Cys77 and Cys263) to alanine residues in the A-domain of full-length matrilin-3 expression constructs (WT and p.V194D) was performed by PCR-based *in vitro* mutagenesis similar to the approach previously used to introduce MED mutations (21).

CHO, HEK 293 and HT1080 cells were cultured as previously described with Dulbecco's Modified Eagle's medium supplemented with 10% FBS (Fisher Scientific), 2 mm l-glutamine (Sigma), 100 U/ml penicillin and 100 mg/ml streptomycin (Biowhittaker), non-essential amino acids and vitamins (Sigma) and antibiotics appropriate to the expression vectors (13,21,30). All cells were incubated at 37°C in humidified air containing 5% CO_2_. CHO and HEK 293 cells were lysed by incubation in RIPA lysis buffer (10 mm Tris, pH 7.4, 150 mm NaCl, 1% sodium deoxycholate, 0.1% SDS) on ice for 15 min. The cell extract was removed, centrifuged at 13 600*g* for 5 min and the supernatant removed for analysis.

### Isolation of total mouse chondrocyte protein

Chondrocytes were isolated from the rib cartilage of pooled litters of 5-day-old mice as described previously (12). Briefly, rib cages were dissected from wild-type (WT) and mutant (mm) mice and treated with 2 mg/ml type 1A collagenase (Sigma) for 1 h. The costal cartilage was then dissected from the rib cage and other connective tissues were removed. The clean cartilage was further treated with collagenase for 3 h, and the collagenase digest passed through a cell strainer (70 μm) and centrifuged for 5 min at 560*g* to pellet the released chondrocytes. The cell pellet was washed twice with PBS and resuspended in 5 ml PBS. Chondrocyte numbers were determined using a haemocytometer and aliquots of 1 × 10^5^ cells prepared. Cell pellets were collected by centrifugation and resuspended in 5X SDS loading buffer.

### SDS–PAGE and western blotting of mouse and cell line protein extract

Twenty micrograms of total cell lysate (CHO or HEK293) or mouse chondrocyte protein extract were separated by SDS–PAGE on 4–12% Bis-Tris gels (Invitrogen). Proteins were transferred to nitrocellulose membranes for western blot analysis. Ponceau staining and/or GAPDH were used to confirm the equal loading of protein prior to probing with antibodies specific to Armet, GAPDH (Abcam), Creld2, GRP78, GRP94, ERp72 (all Santa Cruz Biotechnology), matrilin-3 (R&D), COMP (Genetex) and FLAG (Sigma). Protein bands were visualized by enhanced chemiluminescence (Perkin-Elmer Inc) and quantified using AIDA densitometry software. For the Armet and Creld2 blots using chondrocyte protein extracts, the ‘cell protein aliquots’ from three different biological replicates were analysed in triplicate and quantified for each genotype. Analysis of cell lysates from CHO and HEK293 cells was performed in triplicate on one isolate.

### Immunohistochemical analysis of growth plate cartilage

Limbs from male mice were prepared for IHC as described previously (10). Sections were blocked with goat serum (Dako Cytomation) in PBS/1% bovine serum albumin (BSA) and incubated for 1 h with primary antibodies specific to Armet or Creld2. Sections were then incubated with the appropriate secondary antibody (biotinylated anti-rabbit IgG, Dako Cytomation) in PBS/1% BSA, followed by incubation with ABCcomplex/HRP reagent (Vector laboratories Ltd.) for 30 min. Sections were developed using 3,3′-diaminobenzidine (Dako Cytomation), counterstained with methyl green, dehydrated in EtOH, cleared in xylene and mounted in Vectamount. The sections were imaged using a Zeiss Axiovision microscope.

### Immunocytochemical analysis of MED cell culture models

Fixed cells were permeabilized with 0.2% Triton/PBS for 8 min, washed in PBS, blocked with 2% donkey serum/PBS and incubated with primary antibodies against either matrilin-3 or Armet for 1 h. Cells were then washed with PBS, incubated with the secondary antibody (Alexa Fluor 555 or Fluor 488; Invitrogen) and washed in PBS before mounting in Vectashield medium with DAPI (Vector) and imaged.

### Co-immunoprecipitation of interacting proteins

Co-immunoprecipitation was carried out on HEK-293 cell lysates transfected with matrilin-3 constructs using ANTI-FLAG^®^ M2 affinity gel (Sigma Aldrich, Dorset UK) or on HT1080 cells transfected with COMP constructs using ANTI-GFP sepharose beads (Abcam). Prior to immunoprecipitation, the cells were treated with dithiobis[succinimidyl propionate] to stabilize protein complexes as previously described (30). Cell lysates were prepared by incubation in lysis buffer (50 mm Tris–HCl, pH 7.4, 1% Triton X-100, 150 mm NaCl, 1 mm EDTA). Following removal of the cell monolayer and centrifugation at 13 600*g*, 500 µg of cell lysate was incubated with ANTI-FLAG^®^ M2 affinity gel or ANTI-GFP sepharose beads as per manufacturer's instructions. The resin was collected and washed three times in tris-buffered saline (50 mm Tris–HCl, pH 7.4, 150 mm NaCl), before proteins were eluted with 0.1 m glycine HCl, pH 3.5 for SDS–PAGE and western blotting.

### Mass spectrometry (MS) analysis of protein precipitates

Total protein pools were excised from each lane of an SDS–PAGE gel (run as described above) before being dehydrated, reduced, alkylated and washed. Samples were then digested with trypsin overnight at 37°C and analysed by LC-MS/MS using a NanoAcquity LC (Waters, Manchester, UK) coupled to a LTQ Velos (Thermo Fisher Scientific, Waltham, MA) mass spectrometer. Peptides were concentrated on a pre-column (20 mm × 180 μm i.d, Waters) and were then separated using a gradient from 99% A (0.1% FA in water) and 1% B (0.1% FA in acetonitrile) to 25% B, in 45 min at 200 nl min-1, using a 75 mm × 250 μm i.d. 1.7 μm BEH C18, analytical column (Waters). Peptides were selected for fragmentation automatically by data-dependent analysis.

Data were interrogated using Mascot version 2.2 (Matrix Science, UK) against the UniProt database (version 2011-05) with taxonomy of *Homo sapiens* selected. Mascot search results were validated using Scaffold version 3.3.1 (Proteome Software, Portland, USA) to assign confidence values to peptide/protein matches, where the peptide/protein confidence values of 0.95 and 0.99 were used. Identified proteins were defined as having a number of matched peptide spectra greater than two, and the unweighted spectral count was used as a measure of quantification. Two biological replicates were used in all experiments.

### Generation of Armet and Creld2 substrate-trapping mutants

cDNAs containing the entire coding sequence for Armet and Creld2 were amplified using the PCR. The forward primer included a *Kpn*I site at the 5′ end of the sequence, while the reverse primer included a V5 tag followed by a KDEL sequence, stop codon and a *Xho*I restriction site to aid sub-cloning and identification. The engineered PCR products were ligated into *Kpn*I–*Xho*I-digested pcDNA3.1(+) (Invitrogen). *In vitro* mutagenesis of the N- and C-terminal cysteines in both putative active CXXA sites of Creld2 and Armet was performed by a PCR. For Creld2, these were Cys32 and Cys264 and for Armet Cys33 and Cys154 (Supplementary Material, Figs S5 and S6). Plasmids were linearized with *Ssp*I before transfecting into subconfluent HT1080 human fibroblasts with LipofectAMINE 2000 reagent as described previously. Stable cell lines were selected with G418 for 14 days before colonies were isolated and expanded.

### Identification of putative mixed disulphides using substrate-trapping mutants

HT1080 cells expressing the various forms of Armet-V5, Creld2-V5 (WT, N/C-CXXA, N-CXXA and C-CXXA) and ERp72-V5 (C-CXXA) were grown to confluence under standard conditions and then treated with NEM (25 mm) to preserve mixed disulphides. Cells were lysed in 1% (v/v) Triton X-100, 50 mm Tris–HCl, pH 7.4, 150 mm NaCl, 2 mm ethylenediaminetetraacetic acid and 0.5 mm phenylmethylsulphonyl fluoride and 20 µg cell lysate was separated by SDS–PAGE on 4–12% Bis-Tris gels under either reducing (DTT) or non-reducing conditions. Western blotting was subsequently performed with antibodies specific to the V5 epitope (Invitrogen: 1:5000 dilution).

## SUPPLEMENTARY MATERIAL

Supplementary Material is available at *HMG* online.

*Conflict of Interest statement*. None declared.

## FUNDING

This work was supported by the Wellcome Trust (M.D.B. is the recipient of a Wellcome Trust Senior Research Fellowship in Basic Biomedical Science; Grant 084353/Z/07/Z) and was undertaken in the Wellcome Trust Centre for Cell-Matrix Research (Grant 088785/Z/09/Z) and the Biomolecular Analysis Facility of the Faculty of Life Sciences at the University of Manchester. We thank Dr Katarzyna Pirog for sharing her microarray data from the T585M *Comp* mouse. Funding to pay the Open Access publication charges for this article was provided by the Wellcome Trust.

## Supplementary Material

Supplementary Data
