# Supplementary material for: Armet/Manf and Creld2 are components of a specialized ER stress response provoked by inappropriate formation of disulphide bonds: implications for genetic skeletal diseases
Source: Hum Mol Genet. 2013 Aug 15;22(25):5262–75. doi: 10.1093/hmg/ddt383 (PMC3842181; doi:10.1093/hmg/ddt383)
Supplement: Supplementary Data [file supp_22_25_5262__index.html]

Armet/Manf and Creld2 are components of a specialised ER stress response provoked by inappropriate formation of disulphide bonds: implications for genetic skeletal diseases — Armet/Manf and Creld2 are components of a specialized ER stress response provoked by inappropriate formation of disulphide bonds: implications for genetic skeletal diseases — Armet/Manf and Creld2 are components of a specialized ER stress response provoked by inappropriate formation of disulphide bonds: implications for genetic skeletal diseases — Supplementary Data 

# Armet/Manf and Creld2 are components of a specialized ER stress response provoked by inappropriate formation of disulphide bonds: implications for genetic skeletal diseases

## Supplementary Data

Supplementary Data

**Files in this Data Supplement:**

- Supplementary Data - Pdf file
