# Supplementary material for: Armet/Manf and Creld2 are components of a specialized ER stress response provoked by inappropriate formation of disulphide bonds: implications for genetic skeletal diseases
Source: Hum Mol Genet. 2013 Aug 15;22(25):5262–75. doi: 10.1093/hmg/ddt383 (PMC3842181; doi:10.1093/hmg/ddt383)

Supplemental Figure 1. Armet and Creld2 are up-regulated in chondrocytes from new born and 5 day old V194D *Matn*3 mice [mm] compared to wild type [wt] controls. Equal protein loading was confirmed by Ponceau staining.

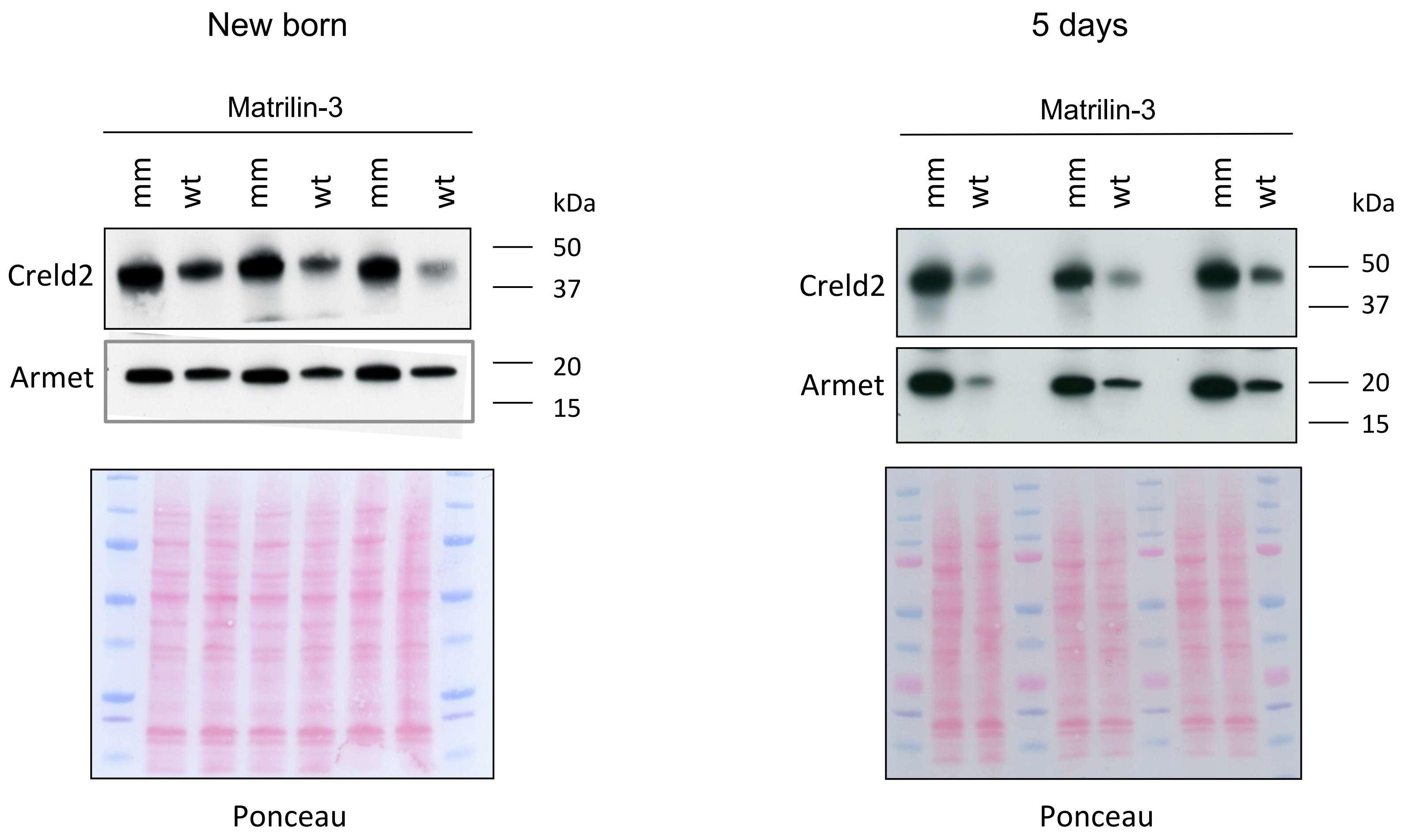

Supplemental Figure 2. Immunohistochemical localisation of ARMET and CRELD2 with V194D matrilin-3 in the cartilage growth plates and femoral heads of 1 week old mice. Key: wild type [WT] and *Matn3* V194D [mm] mice. Scale bar = 200μm.

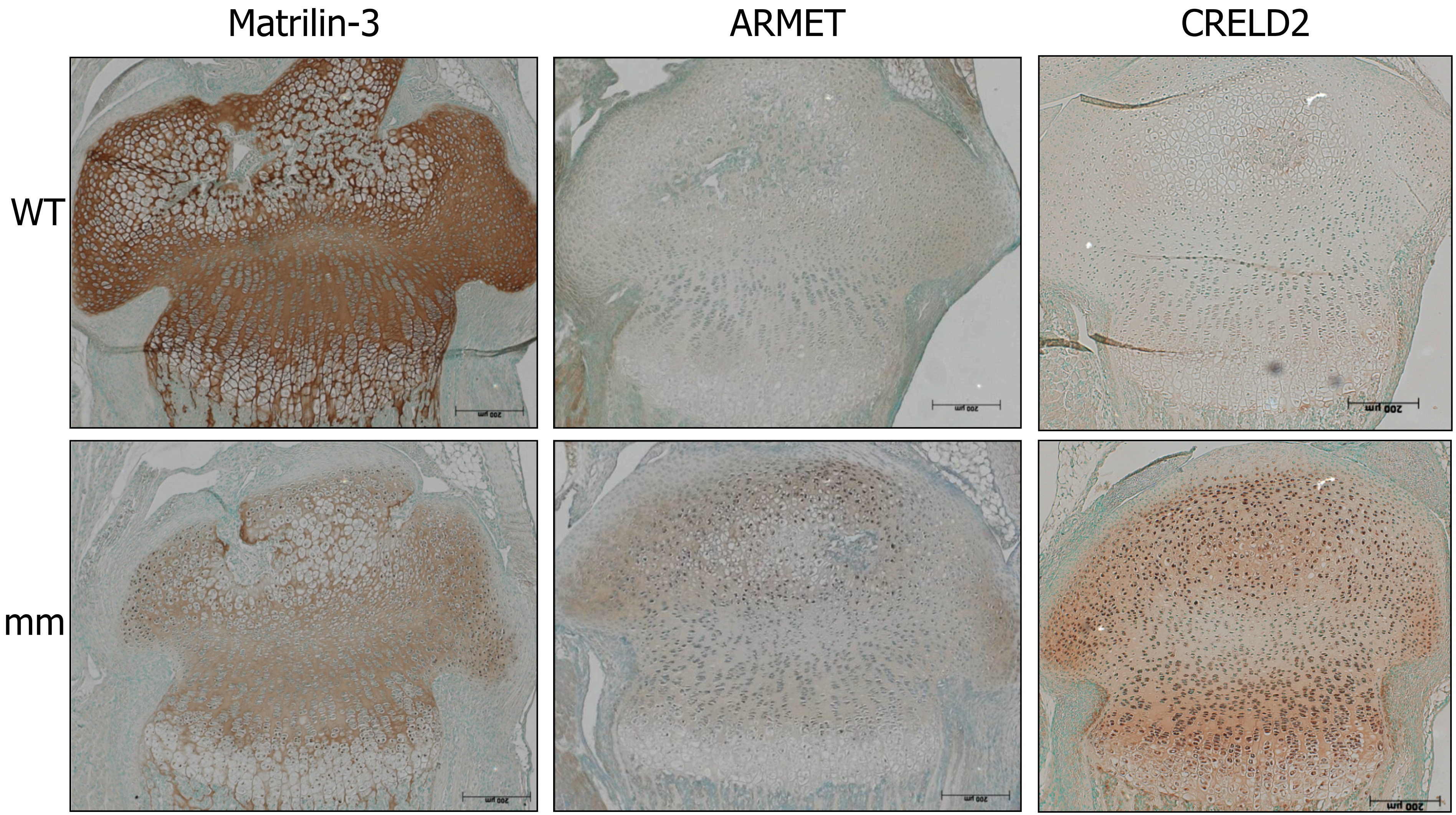

Supplemental Figure 3. IHC analysis of the tibia growth plate shows increased ARMET in the ECM and chondrocytes of *Matn 3* V194D mutant mice [mm] compared to wild type [WT] controls from birth. The different zones of the growth plate are indicated on the top right panel (VIF = vascular invasion fron)t.

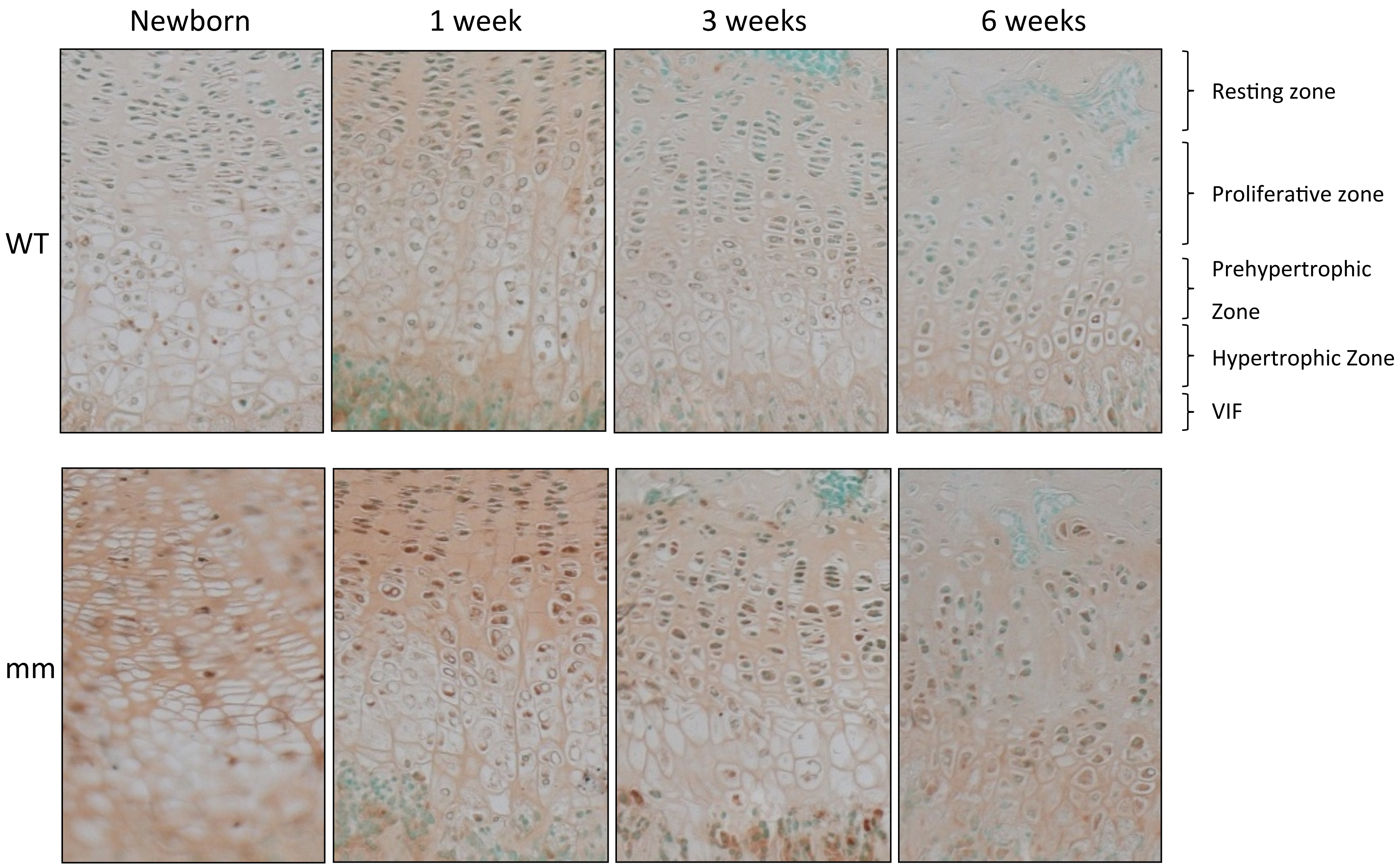

Supplemental Figure 4. IHC analysis of the tibia growth plate shows increased CRELD2 in the ECM and chondrocytes of *Matn3* V194D mutant mice [mm] compared to wild type [WT] controls from birth. The different zones of the growth plate are indicated on the top right panel (VIF = vascular invasion front).

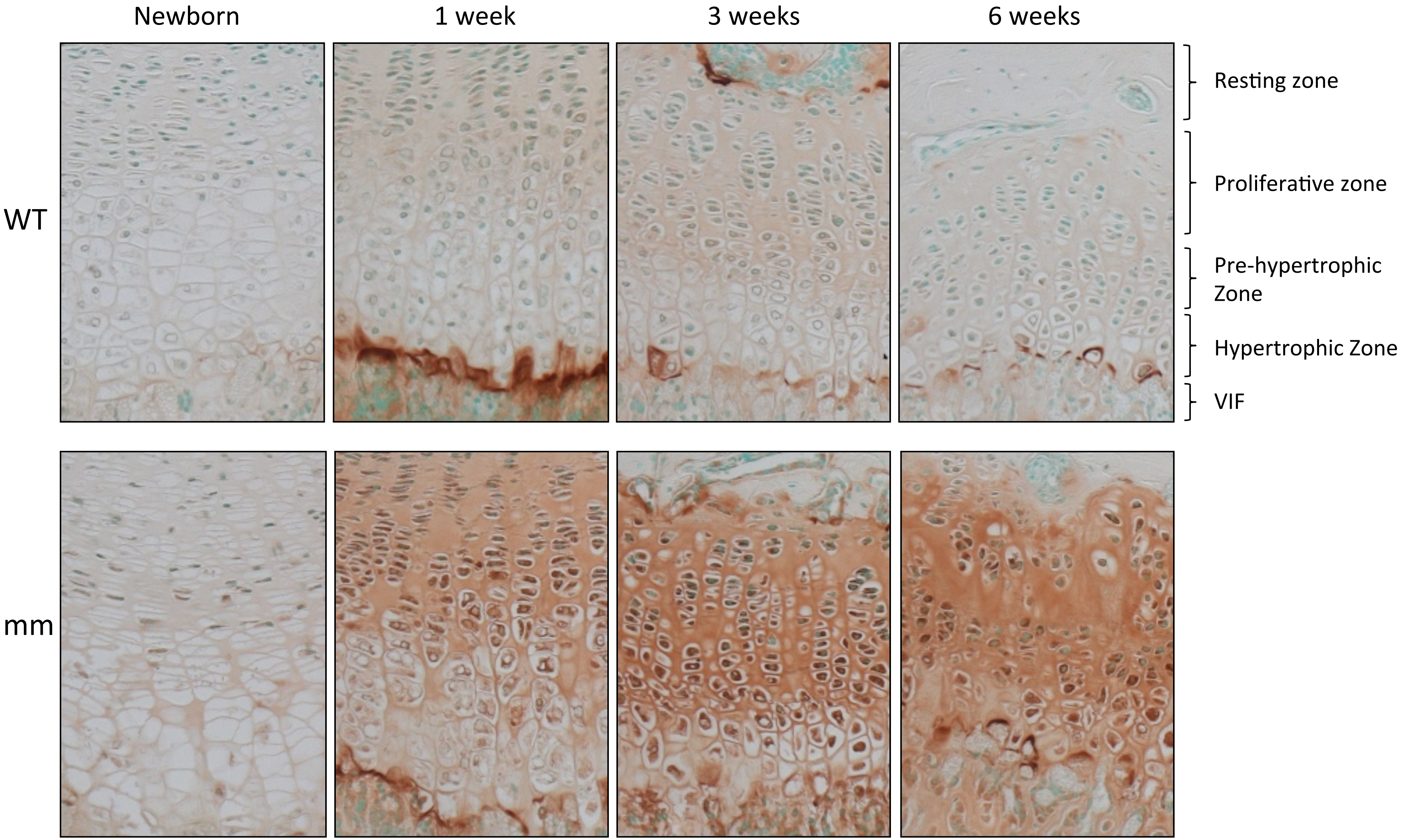

Supplemental Figure 5. There is no up-regulation of CRELD2 in *Comp* models of PSACH-MED (T585M and D469del) compared to *Matn3* V194D in the cartilage growth plates and femoral heads of 1 week old mice. Key: wild type [WT] and mutant [mm] mice. Scale bar = 200μm.

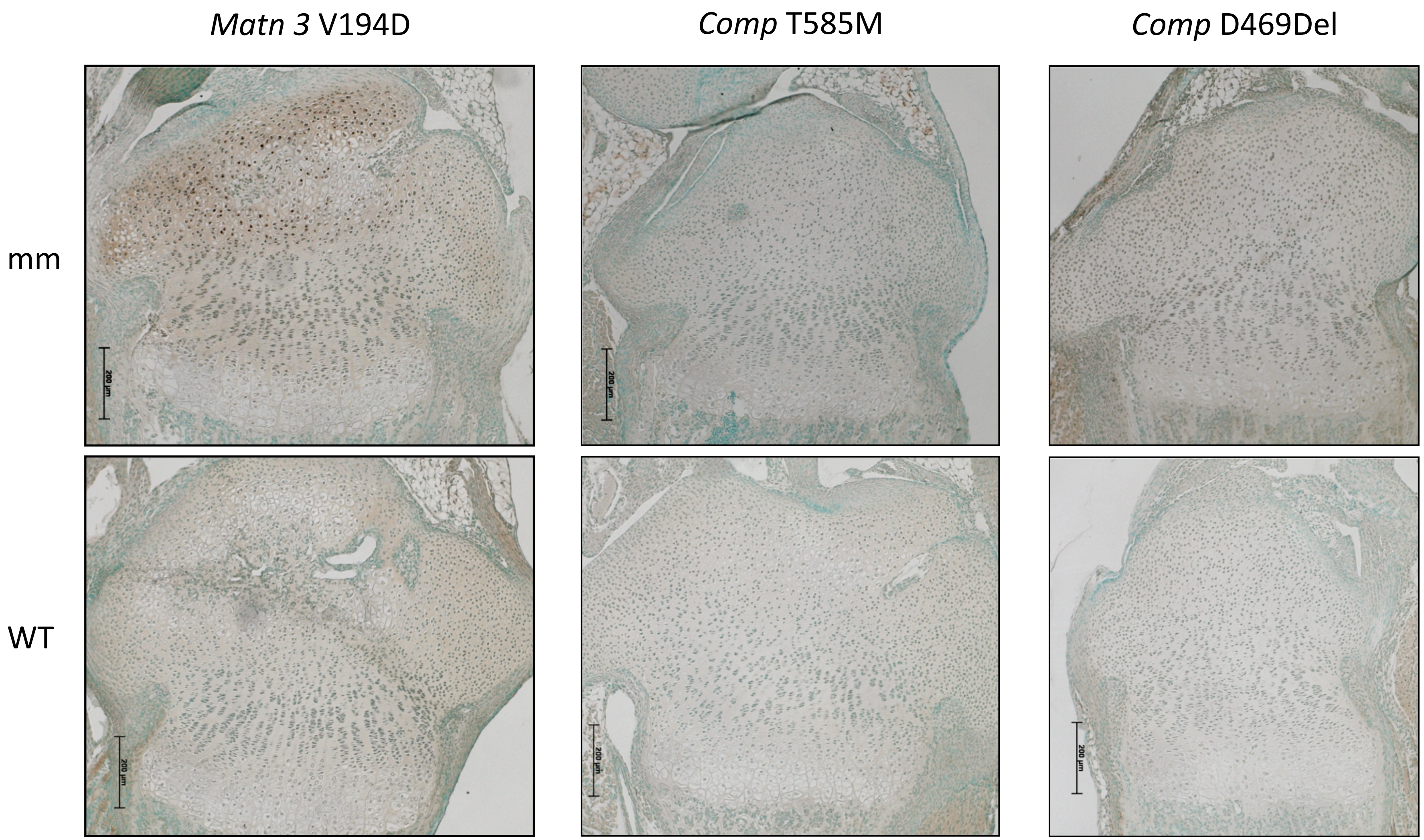

Supplemental Figure 6. CLUSTAL 2.1 multiple sequence alignment between PDI, ARMET and CRELD2. Numbering of amino acid residues is shown on the right with translational start codon (methionine) as 1. The potential CXXC motifs of CRELD2 and ARMET along with the known CXXC motif of PDI are indicated in green and the imperfect KDEL sequences in yellow. Key: * = identical residue; : = conserved residue; . = semi-conserved residue.

```
ARMET           -MRRMWATQGLAVALALSVLPG----------------------SRALRPGDCEVCISYL 37
CRELD2          --MHLLLAAAFGLLLLLPPPGA----------------------VASRKPTMCQRCRTLV 36
PDI             MLRRALLCLAVAALVRADAPEEEDHVLVLRKSNFAEALAAHKYLLVEFYAPWCGHCKALA 60
                   :     ...  :                                  .  *  * :

ARMET           GRFYQD--------------------------------------LKDRD----------- 48
CRELD2          DKFNQG--------------------------------------MANTARKNFGGGNTAW 58
PDI             PEYAKAAGKLKAEGSEIRLAKVDATEESDLAQQYGVRGYPTIKFFRNGDTASPKEYTAGR 120
                 .: :                                       : :

ARMET           ------------------------------------------------------------
CRELD2          EEKTLSKYEFSEIRLLEIMEGLCDSSDFECNQLLEQQEEQLEAWWQTLKKEHPNLFEWFC 118
PDI             EADDIVNWLKKRTGPAATTLPDGAAAESLVESSEVAVIGFFKDVESDSAKQFLQAAEAID 180

ARMET           -VTFSPATIENELIKFCREARGKENRLCY---------------------------YIGA 80
CRELD2          VHTLKACCLPGTYGPDCQECQGGSERPCSGNGYCSGDGSRQGDGSCQCHTGYKGPLCIDC 178
PDI             DIPFGITSNSDVFSKYQLDKDGVVLFKKFDEGRNNFEGEVTKENLLDFIKHNQLPLVIEF 240
                  .:      .       :  *                                   *

ARMET           TDDAATKII----------------NEVSKPLAHHIPVEKICE----------------- 107
CRELD2          TDGFFSLQR----------------NETHSICSACDESCKTCS----------------- 205
PDI             TEQTAPKIFGGEIKTHILLFLPKSVSDYDGKLSNFKTAAESFKGKILFIFIDSDHTDNQR 300
                *:   .                   .:     :      :  .

ARMET           -----KLKKKDS-QICELKYDKQIDLSTVDLKKLRVKELKK------------------I 143
CRELD2          -----GPSNKDC-IQCEVGWAR-VEDACVDVDECAAETSPCSDGQYCE-----NVNGSYT 253
PDI             ILEFFGLKKEECPAVRLITLEEEMTKYKPESEELTAERITEFCHRFLEGKIKPHLMSQEL 360
                       .:::.     :   . :     : .:  .:

ARMET           LDDWGET-----------------------------CKGCAEK----------------- 157
CRELD2          CEDCDST-----------------------------CVGCTGKGPANCKECIAGYTKESG 284
PDI             PEDWDKQPVKVLVGKNFEDVAFDEKKNVFVEFYAPWCGHCKQLAPIWDKLGETYKDHENI 420
                 :* ..                              *  *

ARMET           ------------------------------------------------------------
CRELD2          QCTDIDECSLEEKACKRKNENCYNVPGSFVCVCPEGF----------------------E 322
PDI             VIAKMDSTANEVEAVKVHSFPTLKFFPASADRTVIDYNGERTLDGFKKFLESGGQDGAGD 480

ARMET           ---SDYIRKINELMPKYAPKAASARTDL 182
CRELD2          ETEDACVQTAEGKVTEENPTQPPSREDL 350
PDI             DDDLEDLEEAEEPDMEEDDDQKAVKDEL 508
                      :.  :    :      . : :*
```

Supplemental Figure 7. Substrate trapping mutants were generated by mutating the potential active sites from CXXC to CXXA and included engineered V5 tags and KDEL sequences at C-terminus. *In vitro* mutagenesis of the N- and C-terminal cysteines in both putative active CXXA sites of CRELD2 and ARMET was performed by PCR. For CRELD2 these were Cys32 and Cys264 and for ARMET Cys33 and Cys154.

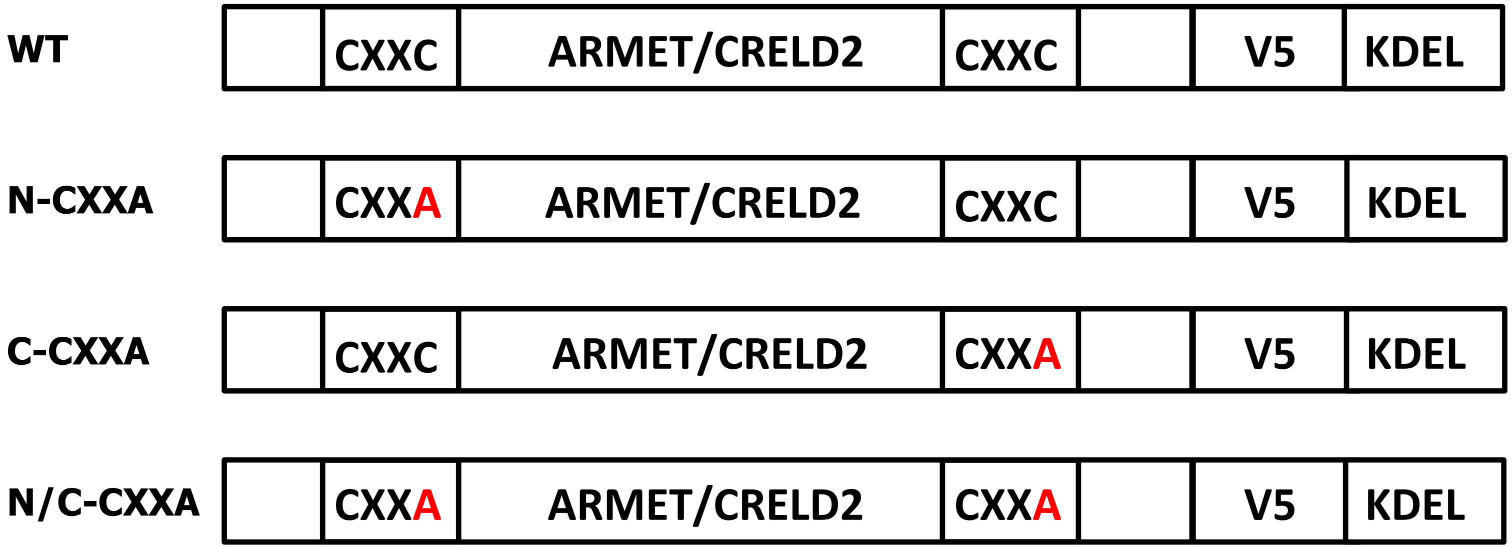

Supplement: Supplementary Data [file supp_ddt383_ddt383supp.pdf]
